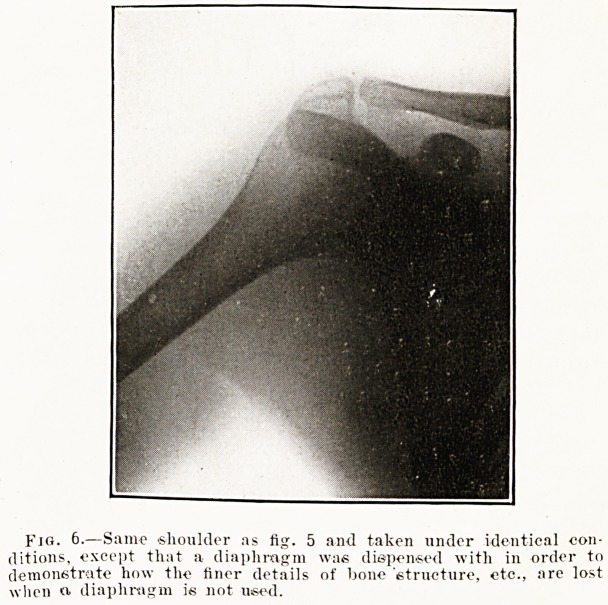# Radiography

**Published:** 1912-12-14

**Authors:** Alfred C. Norman

**Affiliations:** House Surgeon at Durham County Eye Infirmary.


FIG. 1.—Hand and wrist, adult male of average proportions. Exposure—20 milliampere-seconds; direction—postero-anterior; anticathode 18 inches from plate. The image of the small radiometer shows that the penetration was No. 8 Wehnelt—*i.e.* the shadow of the silver strip and that of the aluminium wedge are of the same intensity opposite that number only.

FIG. 2.—Same hand as shown in fig. 1, taken under identical conditions, except that too soft a tube was purposely used. Note the difference. The penetration of the tube was No. 3 Wehnelt, consequently very few rays have passed through the flesh or even through the thin end of the aluminium wedge of the radiometer. The shadow of the needle is lost.

FIG. 3.—Lateral view of dislocated terminal phalanx of little finger, girl, æt. sixteen, 20 milliampere-seconds, No. 5 Wehnelt, 18 inches. The tube was rather too soft to show full detail in the bone, hence the outline of the flesh is well defined.

FIG. 4.—Antero-posterior view of the dislocation shown in fig. 3; the details of exposure were identical in each case; they are shown here to illustrate the importance of radiographing from two points of view.

FIG. 5.—Shoulder, adult male, rather stout, antero-posterior, 80 milliampere-seconds, 21 inches. Taken with cylinder diaphragm having a 2-inch aperture at the tube end. Note the cancellous structure in the head of the humerus and the medullary canal in the shaft.

FIG. 6.—Same shoulder as fig. 5 and taken under identical conditions, except that a diaphragm was dispensed with in order to demonstrate how the finer details of bone structure, etc., are lost when a diaphragm is not used.

### Illustrations for article on "Radiography," by Dr. A. C. Norman.

*See* THE HOSPITAL, *December* 14, 1912, *pages* 299, 300.

### ELECTRICITY IN MODERN MEDICINE.*

### XXIII. Radiography (*Continued*).

By ALFRED C. NORMAN, M.D. Edin., House Surgeon at Durham County Eye Infirmary.

### Penetrating Power of the Rays.

The penetrating power of the rays is by far the most important factor in the whole science of radiography, and, if it be neglected, unsatisfactory results are a foregone conclusion.

Generally speaking, our object must be to select a tube that will give out rays of such penetration that they will be obstructed by the tissue to be investigated, while they pass easily through the surrounding tissues.

If we are investigating the bones, for instance, and we select a tube that is too soft—*i.e.* whose rays are not sufficiently penetrating—the latter will be nearly all obstructed by the flesh, and the resulting radiograph † will be a densely black shadow-outline of the fleshy part, in which the bones can hardly be defined. (See fig. 2.) Such a *shadow-image* might, with accuracy, be termed a *skiagram*. On the other hand, if too hard a tube be used, the rays will pass almost as freely through the bones as through the flesh; consequently the radiograph will have a flat, washed-out appearance, and the delicate structure of the bone will be entirely lost. But if a tube of suitable penetration be selected, the rays will be obstructed exactly in proportion to the densities of the various tissues through which they pass; hence the more delicate anatomical or pathological details will be depicted in the radiograph. Note, for instance, the cancellous tissue of the bones in figs. 1 and 6. Such pictures are more than *skiagrams*; they are *radiographic* charts of the various tissue densities.

In the case of bones that are considerably denser than their surroundings, a small error in adjusting the penetration of the tube will not entirely spoil the result. The heart also allows of some latitude in this respect, because it is so much more opaque than the lungs; but the normal kidney, surrounded as it is by structures that are quite as dense as itself, can seldom be demonstrated even by the most perfect technique. As Bythell and Barclay point out, if the kidneys were situated in the thorax they would be seen, in contrast with the lungs, quite as distinctly as the heart itself. Metallic foreign bodies are, of course, very easily radiographed. The shadow of ordinary glass is usually about as dense as that of the bones, but glass impregnated with lead acetate is one of the most opaque substances we possess, and is largely used in the manufacture of protective shields and the like. Water, the serous fluids, and pus are all very opaque to the $x$-rays; consequently a pleuritic effusion or an empyema is seen in remarkable contrast with the transparent lung tissue. Attempts have been made to diagnose pregnancy in disputed cases by means of the $x$-rays, but the density of the amniotic fluid completely obscures the bones of the fœtus. Renal, ureteral, and vesical calculi can usually be demonstrated in a radiograph, but in some cases of soft calculi considerable technical skill is required to differentiate them from the surrounding tissues.

It only takes a few seconds to observe the penetration of a tube if the small radiometer be kept permanently screwed to a corner of the fluorescent screen. A still better plan is to radiograph the radiometer at the same time as the patient (this was done in figs. 1 and 2) and so make a permanent record of the penetration used. The beginner should carefully note down the penetration of the rays every time he makes an exposure, and he should constantly compare his results until he is able to select a tube that will bring out the utmost detail of the particular tissue he wishes to investigate. Meanwhile he may be glad to have some actual figures to assist him in the selection of a tube of suitable penetration.

First of all, then, let him adopt as his standard a tube with a penetrating power of between 8 and 9 Wehnelt, and let him use this tube, in conjunction with the table of standard exposures already given, for all radiographic purposes except those now to be indicated. For postero-anterior views of the head, a tube of No. 10 Wehnelt should be used; for the pelvis, No. 10; tuberculous foci in the lungs, No. 7; hip-joint, fully No. 9. For the kidney the standard tube should first of all be used, and if this does not show up the calculus, two other radiographs should be made, one with a tube a degree softer and one with a tube a degree harder than the standard. General: For stout adults, a tube rather harder than the standard must be used; for children, a tube rather softer than the standard.

For reasons given on page 246, it is possible to use much softer tubes when radiographing hands and toes, and even the limbs of small children—*provided the exposure be suitably prolonged*. When he has acquired some experience with his standard tube, the worker will find it economical to purchase tubes of about No. 5 penetration, and to use them for these purposes until they become hard enough for standard use.

It is a far more common fault to use too hard a tube than one that is too soft. This is probably due to the fact that all tubes, unless they are grossly overrun, gradually get harder with use, but this is certainly no excuse; modern regenerating devices are so simple to use that any tube, however hard, can at once be brought to the required degree of penetration. Also, a hard tube gives plenty of indication as to its condition, because, owing to the great resistance the current in the secondary circuit is raised to a high voltage and tries to avoid passing through the tube, by sparking to every available conductor and by passing from one wire to another

* Previous articles appeared on Nov. 11, 25, Dec. 9, 30, 1911, Jan. 13, 27, Feb. 17, March 9, 30, April 20, May 4, 25, June 8, July 6, Aug. 3, 17, 31, Sept. 28, Oct. 12, 26, Nov. 9 and 30, 1912.

† This refers to a positive point, not to the negative itself.

in the form of a brush discharge. These phenomena are accompanied by a very audible crackling noise, which serves to warn us that the tube is too hard and is in serious danger of being punctured by a spark.

The penetrating power of the $x$-rays depends upon their wave-length, the wave-length depends upon the velocity with which the cathode stream strikes the anticathode, and the velocity is probably dependent upon three factors: the degree of exhaustion of the tube, the nature of the gas in the tube, and the voltage in the secondary circuit. The writer recently performed the following experiment to demonstrate that voltage, *per se*, has a definite influence upon the penetrating power of the rays. He passed a current of one milliampere through a tube which the radiometer showed to be of No. 6 penetration. He then artificially raised the voltage of the secondary circuit by interposing a six-inch spark-gap in series with the tube, and on again passing a current of one milliampere through the tube the penetration of the latter was found to be No. 8. As soon as the spark-gap was closed the penetration dropped again to No. 6. This probably explains why, when used with the Snook transformer furnishing a lower voltage, a tube of a certain vacuum is less penetrating than when used with an induction coil.

### Some Photographic Considerations.

It is important to stick to one brand of photographic plates if uniform results are desired. Special $x$-ray plates are now made, having an emulsion considerably thicker than could be used for ordinary photographic purposes. The $x$-rays pass right through the thick coating of emulsion, affecting every part of it, and thus allowing us to obtain a negative of good density with a much shorter exposure than could be done with ordinary plates. The writer always uses Ilford $x$-ray plates. He was able to reduce his exposures by nearly one-half from the day he adopted them, and in many thousands of exposures he has never had a faulty result that could be attributed to the plate. The standard table of exposures given on page 246 is based upon results obtained with Ilford plates and developer; if ordinary photographic plates are used the exposures must be doubled. It is equally important that only one brand of developer be used. The writer finds that the metol-hydroquinone formula given on each box of Ilford plates is reliable and very rapid. For general purposes four sizes of plates should be at hand—viz. $6\frac{1}{2}$ by $4\frac{3}{4}$, $8\frac{1}{2}$ by $6\frac{1}{2}$, 10 by 8, and 12 by 10. The stock of plates must be kept as far as possible from the $x$-ray room, because the rays will easily penetrate the cardboard boxes in which they are packed. A dozen of each size should be placed in light-tight envelopes ready for use, and these may be conveniently kept in the $x$-ray room—*provided they are stored in a box lined with sheet-lead one-sixteenth of an inch thick.**

*Numbering the Negative.*—The radiographs may be permanently numbered by placing metal figures on a corner of the envelope and allowing them to remain there while the exposure is made. A set of these figures is quite inexpensive and will last a lifetime.

*To Prevent Distortion* it is essential that the centre of the anticathode be directly over the most important object in the part to be radiographed. In most tube boxes there is a device for accurately centering the anticathode with the diaphragm, so that it only remains for us to centre the diaphragm over the object to be radiographed, and this may be conveniently done by means of a joiner's plumb-bob fastened to the end of a bit of string.

The part must be in close contact with the plate, and must be kept perfectly still during the exposure; a supply of sandbags will be found very convenient for immobilising the limbs. The closer an object is to the plate the sharper will be its image and *vice versâ*. For instance, in a postero-anterior radiograph of the knee the patella is well defined, whereas in an antero-posterior view of the same part the shadow of the patella is difficult to make out. The nearer the tube is to the object the greater will be the magnification of the latter, and the less sharp will be its image. This fact is sometimes utilised in taking lateral views of the facial bones and orbit. The superimposed images of the two sides make accurate interpretation of the radiograph very difficult, but, by bringing the tube nearer, the image of the side next the tube is so magnified and dispersed that the outline of the side in contact with the photographic plate is comparatively sharp.

*Two Points of View.*—Every part that permits of it should be radiographed from two points of view—lateral and either antero-posterior or postero-anterior. There is practically no perspective in a radiograph, hence it is quite easy to miss a fracture or a dislocation, or to locate a foreign body in a totally wrong position, if only one point of view be taken. Figs. 3 and 4 illustrate a dislocated terminal phalanx of the little finger. If fig. 4 alone had been taken it would have been impossible to diagnose a dislocation from the radiograph. Incidentally it may be mentioned that they were taken some years ago with an old type of coil, and that they were the present writer's first attempt at radiography.

*The Use of Diaphragms.*—The extreme value of diaphrams in cutting off secondary rays and thus improving the quality of the radiograph can hardly be overemphasised. Without a diaphragm the finer structure of the tissues cannot possibly be demonstrated. We have seen that secondary rays may arise from three sources: (1) Always from the walls of the tube in front of the anticathode; (2) from almost every part of the tube when there is reverse current; (3) from the tissues of the body through which the rays pass. By using a compresser cylinder-diaphragm (such as fig. 1, p. 99) we are able to cut off most of the secondary radiation from the tube and, by compression, to greatly diminish that which arises in the tissues. Fig. 5 was taken *with* a diaphragm, fig. 6 *without*, other conditions being identical in the two exposures. Note how the structure of the bone is lost in fig. 6; the difference would have been even more marked if there had been any reverse current passing through the tube.

(*To be continued.*)

---

* Lead of about this thickness is technically described as 4 lb. to the square foot.

## Figures and Tables

**Fig. 1. f1:**
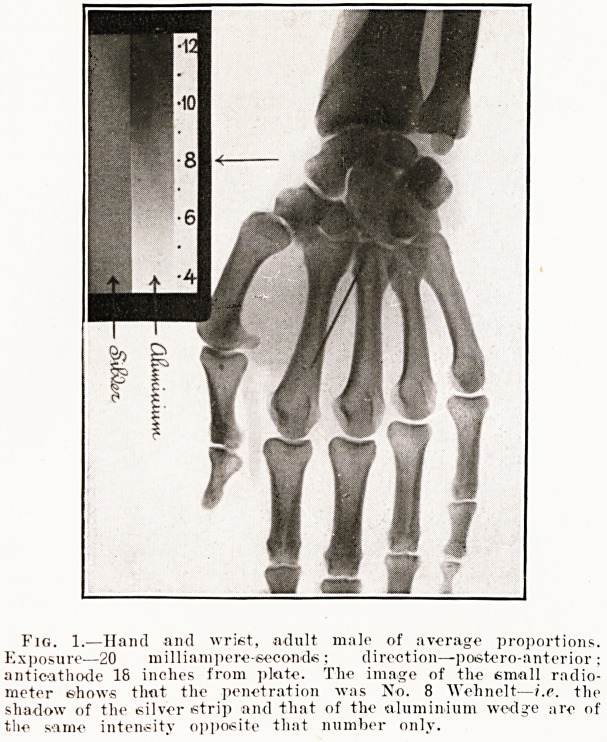


**Fig. 2. f2:**
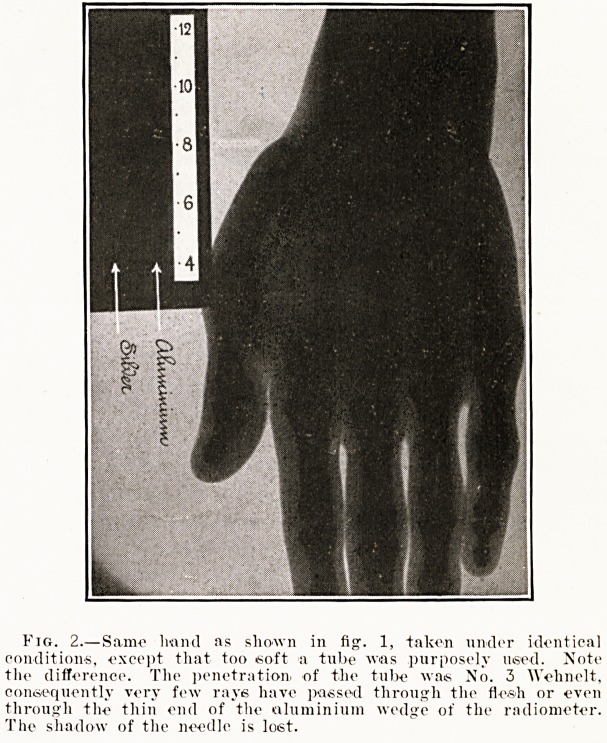


**Fig. 3. f3:**
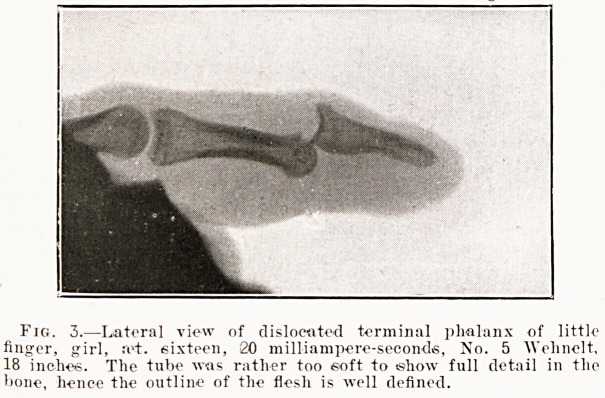


**Fig. 4. f4:**
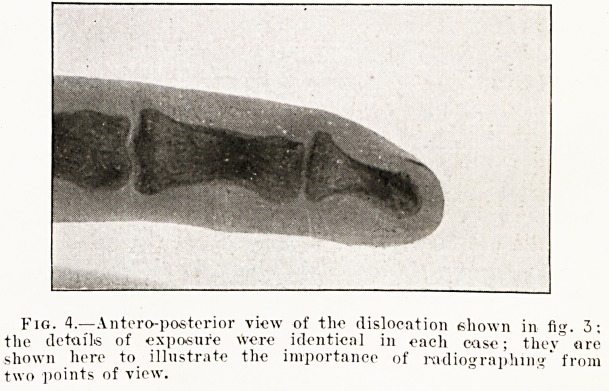


**Fig. 5. f5:**
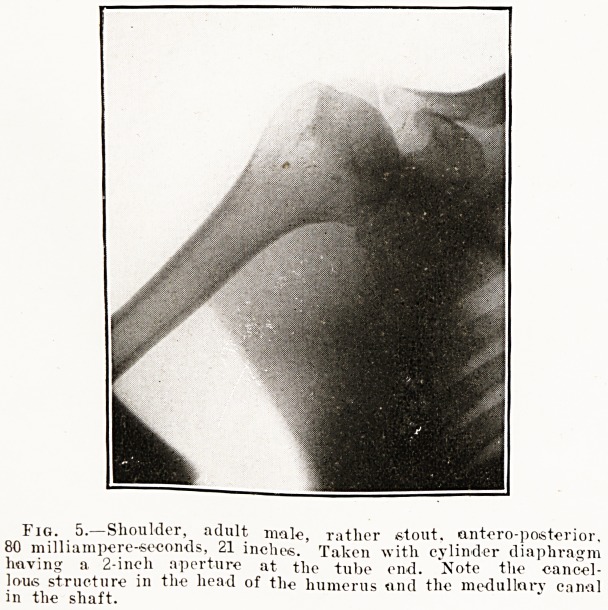


**Fig. 6. f6:**